# Hamming weight and tight constraints of multi-qubit entanglement in terms of unified entropy

**DOI:** 10.1038/s41598-018-30766-2

**Published:** 2018-08-16

**Authors:** Jeong San Kim

**Affiliations:** Department of Applied Mathematics and Institute of Natural Sciences, Kyung Hee University, Yongin-si, Gyeonggi-do 446-701 Korea

## Abstract

We establish a characterization of multi-qubit entanglement constraints in terms of non-negative power of entanglement measures based on unified-(*q*, *s*) entropy. Using the Hamming weight of the binary vector related with the distribution of subsystems, we establish a class of tight monogamy inequalities of multi-qubit entanglement based on the *α*th-power of unified-(*q*, *s*) entanglement for *α* ≥ 1. For 0 ≤ *β* ≤ 1, we establish a class of tight polygamy inequalities of multi-qubit entanglement in terms of the *β*th-power of unified-(*q*, *s*) entanglement of assistance. Thus our results characterize the monogamy and polygamy of multi-qubit entanglement for the full range of non-negative power of unified entanglement.

## Introduction

Quantum entanglement is a quintessential feature of quantum mechanics revealing the fundamental insights into the nature of quantum correlations. One distinct property of quantum entanglement without any classical counterpart is its limited shareability in multi-party quantum systems, known as the *monogamy of entanglement* (MoE)^1,2^. MoE is the fundamental ingredient in many quantum information processing tasks such as quantum cryptography^3,4^, and even in condensed-matter physics such as the *N*-representability problem for fermions^5^.

Mathematically, MoE is characterized in forms of an inequality, namely *monogamy inequality*. The first monogamy inequality was established in three-qubit systems by Coffman-Kundu-Wootters (CKW) as

$$\tau ({\rho }_{A|BC})\ge \tau ({\rho }_{A|B})+\tau ({\rho }_{A|C})$$

for a three-qubit quantum state *ρ*_*ABC*_ with its two-qubit reduced density matrices *ρ*_*AB*_ = tr_*C*_*ρ*_*ABC*_ and *ρ*_*AC*_ = tr_*B*_*ρ*_*ABC*_, where *τ*(*ρ*_*A*|*BC*_) is the bipartite entanglement between subsystems *A* and *BC*, quantified by *tangle* and *τ*(*ρ*_*A*|*B*_) and *τ*(*ρ*_*A*|*C*_) are the tangle between *A* and *B* and between *A* and *C*, respectively^6^. The tangle of a bipartite pure state $|{\psi }_{AB}\rangle$ is defined as $\tau ({|\psi \rangle }_{A|B})=2(1-{\rm{tr}}{\rho }_{A}^{2})$ where ${\rho }_{A}={{\rm{tr}}}_{B}{|\psi \rangle }_{AB}\langle \psi |$ is the reduced density matrix of ${|\psi \rangle }_{AB}$ onto the subsystem *A*. For a bipartite mixed state *ρ*_*AB*_, its tangle is defined as $\tau ({\rho }_{A|B})=\,{\rm{\min }}\,{\sum }_{i}\,{p}_{i}\tau ({|{\psi }_{i}\rangle }_{A|B})$ where the minimum is taken over all possible pure-state decompositions of ${\rho }_{AB}={\sum }_{i}\,{p}_{i}{|{\psi }_{i}\rangle }_{AB}\langle {\psi }_{i}|$^6^. Later, three-qubit CKW inequality was generalized for arbitrary multi-qubit systems^7^ and some cases of multi-party, higher-dimensional quantum systems more than qubits in terms of various bipartite entanglement measures^8–11^.

Using the *assisted entanglement* that is a dual amount to bipartite entanglement measures, a dually monogamous (thus polygamous) property of multi-party entanglement was also established; for a three-qubit state *ρ*_*ABC*_, a *polygamy inequality* was proposed as

$${\tau }^{a}({\rho }_{A|BC})\le {\tau }^{a}({\rho }_{A|B})+{\tau }^{a}({\rho }_{A|C}),$$

where ${\tau }^{a}({\rho }_{A|B})=\,{\rm{\max }}\,{\sum }_{i}\,{p}_{i}\tau ({|{\psi }_{i}\rangle }_{A|B})$ is the tangle of assistance whose maximum is taken over all possible pure-state decompositions of ${\rho }_{AB}={\sum }_{i}\,{p}_{i}{|{\psi }_{i}\rangle }_{AB}\langle {\psi }_{i}|$^12,13^. Later, this tangle-based polygamy inequality of entanglement was generalized into multi-qubit systems as well as some class of higher-dimensional quantum systems using various entropic entanglement measures^10,14,15^. General polygamy inequalities of entanglement were also established in arbitrary dimensional multi-party quantum systems^16,17^.

Entanglement is an intrinsic nature of quantum states in composite systems that cannot be realized by local operations and classical communications (LOCC). Thus it is natural to classify entangled states by means of their interconvertibility under LOCC with non-zero probability, that is, stochastic LOCC (SLOCC). Unlike bipartite entanglement, multi-party quantum entanglement is known to have several inequivalent classes by means of SLOCC interconvertibility^18^. For example, it is known that genuine three-qubit pure entanglement has two inequivalent classes, *Greenberger*-*Horne*-*Zeilinger* (GHZ) class^19^ and W class^18^.

Although GHZ and W classes are both considered as the genuine three-qubit entanglement, these inequivalent classes reveal different characters; W-class states assume the maximum expected amount of two-qubit entanglement when one qubit is traced out, while GHZ-class states loose most two-qubit entanglement. This different characteristics can also be investigated in terms of entanglement monogamy and polygamy, as CKW and its dual inequalities for three-qubit systems are saturated by the W-class states, while each inequality can assume the largest differences between both sides for the GHZ-class states.

The saturation of CKW and its dual inequalities for the W-class states can be physically interpreted as a complete characterization genuine three-qubit (multi-party) entanglement in terms of two-qubit (bipartite) ones within it, whereas GHZ class has too fragile two-qubit ones to have such interpretation. Thus, not only a distinct phenomena in multi-party quantum systems, entanglement monogamy and polygamy can be used as an efficient tool to characterize multi-party quantum entanglement among different classes. However, three-qubit CKW inequality based on tangle is no more valid in higher-dimensional systems due to the existence of counterexamples in $3\otimes 3\otimes 3$ and even in $3\otimes 2\otimes 2$ quantum systems^8,20^. Thus it is an important task to have proper bipartite entanglement quantifications, besides tangle, showing tight monogamy and polygamy inequalities for efficient characterization of multi-party entanglements from different classes in multi-party and even in high-dimensional quantum systems.

Recently, a new class of monogamy inequalities using the *α*th-power of entanglement measures were proposed; for $\alpha \ge \sqrt{2}$, the *α*th-power of entanglement of formation was shown to be used to establish a class of monogamy inequality of multi-qubit entanglement^21^, and a similar result was proposed using the *α*th-power of concurrence for *α* ≥ 2^21^. Later, tighter monogamy and polygamy inequalities of entanglement using non-negative power of concurrence and square of convex-roof extended negativity were also proposed for multi-qubit systems^22,23^.

Here, we provide a full characterization of multi-qubit entanglement monogamy and polygamy constraints in terms of non-negative power of entanglement measures based on unified entropy^24,25^. Using the Hamming weight of the binary vector related with the distribution of subsystems, we establish a class of monogamy inequalities of multi-qubit entanglement based on the *α*th-power of unified-(*q*, *s*) entanglement^11^ for *α* ≥ 1. For 0 ≤ *β* ≤ 1, we establish a class of polygamy inequalities of multi-qubit entanglement in terms of the *β*th-power of unified-(*q*, *s*) entanglement of assistance^15^. Our results of monogamy and polygamy inequalities established here hold in a tighter way than other multi-qubit entanglement inequalities provided so far. Moreover, our new class of monogamy inequalities are also valid for the counterexamples of CKW monogamy inequality in higher-dimensional systems more than qubits.

This paper is organized as follows: First, we review the definitions of unified entropy, unified-(*q*, *s*) entanglement and unified-(*q*, *s*) entanglement of assistance as well as multi-qubit monogamy and polygamy inequalities in terms of unified entanglements. After providing notations and definitions about binary vectors and Hamming weight, we establish a class of tight monogamy inequalities in multi-qubit system based on the *α*th-power of unified-(*q*, *s*) entanglement for *α* ≥ 1. We further establish a class of tight polygamy inequalities of multi-qubit entanglement in terms of the *β*th-power of unified-(*q*, *s*) entanglement of assistance for 0 ≤ *β* ≤ 1. Finally, we summarize our results.

## Results

### Unified entropy and multi-qubit entanglement constraints

For *q*, *s* ≥ 0 with *q* ≠ 1 and *s* ≠ 0, unified-(*q*, *s*) entropy of a quantum state *ρ* is defined as^24,25^,

1 $${S}_{q,s}(\rho )\,:\,=\frac{1}{(1-q)s}[{({\rm{tr}}{\rho }^{q})}^{s}-1].$$

Although unified-(*q*, *s*) entropy has a singularity at *s* = 0, it converges to Rényi-*q* entropy as *s* tends to 0^26,27^. We also note that unified-(*q*, *s*) entropy converges to Tsallis-*q* entropy^28^ when *s* tends to 1, and for any nonnegative *s*, unified-(*q*, *s*) entropy converges to von Neumann entropy as *q* tends to 1,

2 $$\mathop{\mathrm{lim}}\limits_{q\to 1}\,{S}_{q,s}(\rho )=-\,{\rm{tr}}\,\rho \,\mathrm{log}\,\rho =\,:S(\rho ).$$

Using unified-(*q*, *s*) entropy in Eq. (1), a two-parameter class of bipartite entanglement measures was introduced; for a bipartite pure state ${|\psi \rangle }_{AB}$, its *unified*-(*q*, *s*) *entanglement* (UE)^11^ is

3 $${E}_{q,s}({|\psi \rangle }_{A|B})\,:\,={S}_{q,s}({\rho }_{A}),$$

for each *q*, *s* ≥ 0 where ${\rho }_{A}={{\rm{tr}}}_{B}{|\psi \rangle }_{AB}\langle \psi |$ is the reduced density matrix of ${|\psi \rangle }_{AB}$ onto subsystem *A*. For a bipartite mixed state *ρ*_*AB*_, its UE is

4 $${E}_{q,s}({\rho }_{A|B})\,:\,=\,{\rm{\min }}\,\sum _{i}\,{p}_{i}{E}_{q,s}({|{\psi }_{i}\rangle }_{A|B}),$$

where the minimum is taken over all possible pure state decompositions of ${\rho }_{AB}={\sum }_{i}\,{p}_{i}{|{\psi }_{i}\rangle }_{AB}\langle {\psi }_{i}|$. As a dual concept to UE, *unified*-(*q*, *s*) *entanglement of assistance* (UEoA) was also introduced as

5 $${E}_{q,s}^{a}({\rho }_{A|B})\,:\,=\,{\rm{\max }}\,\sum _{i}\,{p}_{i}{E}_{q,s}({|{\psi }_{i}\rangle }_{A|B}),$$

for *q*, *s* ≥ 0 where the maximum is taken over all possible pure state decompositions of *ρ*_*AB*_^15^.

Due to the continuity of UE in Eq. (4) with respect to the parameters *q* and *s*, UE reduces to *Rényi*-*q entanglement* (RE)^9^ as *s* tends to 0, and it also reduces to *Tsallis*-*q entanglement* (TE)^10^ as *s* tends to 1. For any nonnegative *s*, UE converges to *entanglement of formation* (EoF) as *q* tends to 1,

6 $$\mathop{\mathrm{lim}}\limits_{q\to 1}\,{E}_{q,s}({\rho }_{A|B})={E}_{f}({\rho }_{A|B}),$$

therefore UE is one of the most general classes of bipartite entanglement measures including the classes of Rényi and Tsallis entanglements and EoF as special cases^11^. Similarly, the continuity of UEoA in Eq. (5) with respect to the parameters *q* and *s* assures that UEoA reduces to *Rényi*-*q entanglement of assistance* (REoA)^9^ and *Tsallis*-*q entanglement of assistance* (TEoA)^10^ when *s* tends to 0 or 1 respectively. For any nonnegative *s*, UEoA reduces to *entanglement of assistance* (EoA)

7 $$\mathop{\mathrm{lim}}\limits_{q\to 1}\,{E}_{q,s}^{a}({\rho }_{AB})={E}^{a}({\rho }_{AB}),$$

when *q* tends to 1^15^.

Using UE as the bipartite entanglement measure, a two-parameter class of monogamy inequalities of multi-qubit entanglement was established^11^; for *q* ≥ 2, 0 ≤ *s* ≤ 1 and *qs* ≤ 3, we have

8 $${E}_{q,s}({\rho }_{{A}_{1}|{A}_{2}\cdots {A}_{N}})\ge \sum _{i=2}^{N}\,{E}_{q,s}({\rho }_{{A}_{1}|{A}_{i}})$$

for any multi-qubit state ${\rho }_{{A}_{1}\cdots {A}_{N}}$ where ${E}_{q,s}({\rho }_{{A}_{1}|{A}_{2}\cdots {A}_{N}})$ is the UE of ${\rho }_{{A}_{1}{A}_{2}\cdots {A}_{N}}$ with respect to the bipartition between *A*_1_ and *A*_2_ $\cdots$ *A*_*N*_, and ${E}_{q,s}({\rho }_{{A}_{1}|{A}_{i}})$ is the unified-(*q*, *s*) entanglement of the reduced density matrix ${\rho }_{{A}_{1}{A}_{i}}$ for each *i* = 2, …, *N*.

Later, it was shown that unified entropy can also be used to establish a class of polygamy inequalities of multi-qubit entanglement^15^; for 1 ≤ *q* ≤ 2 and −*q*^2^ + 4*q* − 3 ≤ *s* ≤ 1, we have

9 $${E}_{q,s}^{a}({\rho }_{{A}_{1}|{A}_{2}\cdots {A}_{N}})\le \sum _{i=2}^{N}\,{E}_{q,s}^{a}({\rho }_{{A}_{1}|{A}_{i}})$$

for any multi-qubit state ${\rho }_{{A}_{1}\cdots {A}_{N}}$ where ${E}_{q,s}^{a}({\rho }_{{A}_{1}|{A}_{2}\cdots {A}_{N}})$ is the UEoA of ${\rho }_{{A}_{1}{A}_{2}\cdots {A}_{N}}$ with respect to the bipartition between *A*_1_ and *A*_2_ $\cdots$ *A*_*N*_, and ${E}_{q,s}^{a}({\rho }_{{A}_{1}|{A}_{i}})$ is the UEoA of ${\rho }_{{A}_{1}{A}_{i}}$ for *i* = 2, …, *N*.

### Tight monogamy constraints of multi-qubit entanglement in terms of unified entanglement

In this section, we establish a class of tight monogamy inequalities of multi-qubit entanglement using the *α*’th power of UE. Before we present our main results, we first provide some notations, definitions and a lemma, which are useful throughout this paper.

For any nonnegative integer *j* whose binary expansion is

10 $$j=\sum _{i=0}^{n-1}\,{j}_{i}{2}^{i}$$

where ${\mathrm{log}}_{2}\,j\le n$ and *j*_*i*_ ∈ {0, 1} for *i* = 0, …, *n* − 1, we can always define a unique binary vector associated with *j*, which is defined as

11 $$\overrightarrow{j}=({j}_{0},{j}_{1},\ldots ,{j}_{n-1}).$$

For the binary vector $\overrightarrow{j}$ in Eq. (11), its *Hamming weight*, ${\omega }_{H}(\overrightarrow{j})$, is the number of 1’s in its coordinates^29^. We also provide the following lemma whose proof is easily obtained by some straightforward calculus.

#### **Lemma 1**.

*For* $x\in [0,\,1]$ *and nonnegative real numbers α*, *β*, *we have*

12 $${(1+x)}^{\alpha }\ge 1+\alpha {x}^{\alpha }$$

*for α* ≥ 1, *and*

13 $${(1+x)}^{\beta }\le 1+\beta {x}^{\beta }$$

*for* 0 ≤ *β* ≤ 1.

Now we provide our first result, which states that a class of tight monogamy inequalities of multi-qubit entanglement can be established using the *α*-powered UE and the Hamming weight of the binary vector related with the distribution of subsystems.

#### **Theorem 2**.

*For α* ≥ 1, *q* ≥ 2 *and* 0 ≤ *s* ≤ 1, *qs* ≤ 3, *we have*

14 $${({E}_{q,s}({\rho }_{A|{B}_{0}{B}_{1}\cdots {B}_{N-1}}))}^{\alpha }\ge \sum _{j=0}^{N-1}\,{\alpha }^{{\omega }_{H}(\overrightarrow{j})}{({E}_{q,s}({\rho }_{A|{B}_{j}}))}^{\alpha },$$

*for any multi-qubit state* ${\rho }_{A{B}_{0}\cdots {B}_{N-1}}$ *where* $\overrightarrow{j}=({j}_{0},\ldots ,{j}_{n-1})$ *is the vector from the binary representation of j and* ${\omega }_{H}(\overrightarrow{j})$ *is the Hamming weight of* $\overrightarrow{j}$.

#### *Proof*.

Without loss of generality, we may assume that the ordering of the qubit subsystems *B*_0_, …, *B*_*N*−1_ satisfies

15 $${E}_{q,s}({\rho }_{A|{B}_{j}})\ge {E}_{q,s}({\rho }_{A|{B}_{j+1}})\ge 0$$

for each *j* = 0, …, *N* − 2 by reordering and relabeling them, if necessary. From the monotonicity of the function *f*(*x*) = *x*^*α*^ for *α* ≥ 1 and the UE-based monogamy inequality of multi-qubit entanglement in (8), we have

16 $${({E}_{q,s}({\rho }_{A|{B}_{0}{B}_{1}\cdots {B}_{N-1}}))}^{\alpha }\ge {(\sum _{j=0}^{N-1}{E}_{q,s}({\rho }_{A|{B}_{j}}))}^{\alpha },$$

which makes it feasible to prove the theorem by showing

17 $${(\sum _{j=0}^{N-1}{E}_{q,s}({\rho }_{A|{B}_{j}}))}^{\alpha }\ge \sum _{j=0}^{N-1}\,{\alpha }^{{\omega }_{H}(\overleftarrow{j})}{({E}_{q,s}({\rho }_{A|{B}_{j}}))}^{\alpha }.$$

We first prove Inequality (17) for the case that *N* = 2^*n*^, a power of 2, by using mathematical induction on *n*, and extend the result for any positive integer *N*.

For *n* = 1 and a three-qubit state ${\rho }_{A{B}_{0}{B}_{1}}$ with two-qubit rduced density matrices ${\rho }_{A{B}_{0}}$ and ${\rho }_{A{B}_{1}}$, we have

18 $${({E}_{q,s}({\rho }_{A|{B}_{0}})+{E}_{q,s}({\rho }_{A|{B}_{1}}))}^{\alpha }={({E}_{q,s}({\rho }_{A|{B}_{0}}))}^{\alpha }\,{(1+\frac{{E}_{q,s}({\rho }_{A|{B}_{1}})}{{E}_{q,s}({\rho }_{A|{B}_{0}})})}^{\alpha },$$

where Inequalities (12) and (15) implies

19 $${(1+\frac{{E}_{q,s}({\rho }_{A|{B}_{1}})}{{E}_{q,s}({\rho }_{A|{B}_{0}})})}^{\alpha }\ge 1+\alpha {(\frac{{E}_{q,s}({\rho }_{A|{B}_{1}})}{{E}_{q,s}({\rho }_{A|{B}_{0}})})}^{\alpha }.$$

From Eq. (18) and Inequality (19), we have

20 $${({E}_{q,s}({\rho }_{A|{B}_{0}})+{E}_{q,s}({\rho }_{A|{B}_{1}}))}^{\alpha }\ge {({E}_{q,s}({\rho }_{A|{B}_{0}}))}^{\alpha }+\alpha {({E}_{q,s}({\rho }_{A|{B}_{1}}))}^{\alpha },$$

which recovers Inequality (17) for *n* = 1.

Now let us assume Inequality (17) is true for *N* = 2^*n*−1^ with *n* ≥ 2, and consider the case that *N* = 2^*n*^. For an (*N* + 1)-qubit state ${\rho }_{A{B}_{0}{B}_{1}\cdots {B}_{N-1}}$ with its two-qubit reduced density matrices ${\rho }_{A{B}_{j}}$ with *j* = 0, …, *N* − 1, we have

21 $${(\sum _{j=0}^{N-1}{E}_{q,s}({\rho }_{A|{B}_{j}}))}^{\alpha }={(\sum _{j=0}^{{2}^{n-1}-1}{E}_{q,s}({\rho }_{A|{B}_{j}}))}^{\alpha }{(1+\frac{{\sum }_{j={2}^{n-1}}^{{2}^{n}-1}{E}_{q,s}({\rho }_{A|{B}_{j}})}{{\sum }_{j=0}^{{2}^{n-1}-1}{E}_{q,s}({\rho }_{A|{B}_{j}})})}^{\alpha }.$$

Because the ordering of subsystems in Inequality (15) implies

22 $$0\le \frac{{\sum }_{j={2}^{n-1}}^{{2}^{n}-1}\,{E}_{q,s}({\rho }_{A|{B}_{j}})}{{\sum }_{j=0}^{{2}^{n-1}-1}\,{E}_{q,s}({\rho }_{A|{B}_{j}})}\le 1,$$

Inequality (12) and Eq. (21) lead us to

23 $${(\sum _{j=0}^{N-1}{E}_{q,s}({\rho }_{A|{B}_{j}}))}^{\alpha }\ge {(\sum _{j=0}^{{2}^{n-1}-1}{E}_{q,s}({\rho }_{A|{B}_{j}}))}^{\alpha }+\alpha {(\sum _{j={2}^{n-1}}^{{2}^{n}-1}{E}_{q,s}({\rho }_{A|{B}_{j}}))}^{\alpha }.$$

From the induction hypothesis, we have

24 $${(\sum _{j=0}^{{2}^{n-1}-1}{E}_{q,s}({\rho }_{A|{B}_{j}}))}^{\alpha }\ge \sum _{j=0}^{{2}^{n-1}-1}\,{\alpha }^{{\omega }_{H}(\overrightarrow{j})}{({E}_{q,s}({\rho }_{A|{B}_{j}}))}^{\alpha }.$$

Moreover, the last summation in Inequality (23) is also a summation of 2^*n*−1^ terms starting from *j* = 2^*n*−1^ to *j* = 2^*n*^ − 1. Thus, (after possible indexing and reindexing subsystems, if necessary) the induction hypothesis also leads us to

25 $${(\sum _{j{\mathrm{=2}}^{n-1}}^{{2}^{n}-1}{E}_{q,s}({\rho }_{A|{B}_{j}}))}^{\alpha }\ge \sum _{j{\mathrm{=2}}^{n-1}}^{{2}^{n}-1}\,{\alpha }^{{\omega }_{H}(\overrightarrow{j})-1}{({E}_{q,s}({\rho }_{A|{B}_{j}}))}^{\alpha }\mathrm{.}$$

Inequalities (23), (24) and (25) recover Inequality (17) for *N* = 2^*n*^.

Now let us consider an arbitrary positive integer *N* and a (*N* + 1)-qubit state ${\rho }_{A{B}_{0}{B}_{1}\cdots {B}_{N-1}}$. We first note that we can always consider a power of 2, which is an upper bound of *N*, that is, 0 ≤ *N* ≤ 2^*n*^ for some *n*. We also consider a (2^*n*^ + 1)-qubit state

26 $${{\rm{\Gamma }}}_{A{B}_{0}{B}_{1}\cdots {B}_{{2}^{n}-1}}={\rho }_{A{B}_{0}{B}_{1}\cdots {B}_{N-1}}\otimes {\sigma }_{{B}_{N}\cdots {B}_{{2}^{n}-1}},$$

which is a product of ${\rho }_{A{B}_{0}{B}_{1}\cdots {B}_{N-1}}$ and an arbitrary (2^*n*^ − *N*)-qubit state ${\sigma }_{{B}_{N}\cdots {B}_{{2}^{n}-1}}$.

Because ${{\rm{\Gamma }}}_{A{B}_{0}{B}_{1}\cdots {B}_{{2}^{n}-1}}$ is a (2^*n*^ + 1)-qubit state, we have

27 $${({E}_{q,s}({{\rm{\Gamma }}}_{A|{B}_{0}{B}_{1}\cdots {B}_{{2}^{n}-1}}))}^{\alpha }\ge \sum _{j=0}^{{2}^{n}-1}\,{\alpha }^{{\omega }_{H}(\overrightarrow{j})}{({E}_{q,s}({{\rm{\Gamma }}}_{A|{B}_{j}}))}^{\alpha },$$

where ${{\rm{\Gamma }}}_{A{B}_{j}}$ is the two-qubit reduced density matric of ${{\rm{\Gamma }}}_{A{B}_{0}{B}_{1}\cdots {B}_{{2}^{n}-1}}$ for each *j* = 0, …, 2^*n*^ − 1. On the other hand, the separability of ${{\rm{\Gamma }}}_{A{B}_{0}{B}_{1}\cdots {B}_{{2}^{n}-1}}$ with respect to the bipartition between *AB*_0_ $\cdots$ *B*_*N*−1_ and ${B}_{N}\cdots {B}_{{2}^{n}-1}$ assures

28 $${E}_{q,s}({{\rm{\Gamma }}}_{A|{B}_{0}{B}_{1}\cdots {B}_{{2}^{n}-1}})={E}_{q,s}({\rho }_{A|{B}_{0}{B}_{1}\cdots {B}_{N-1}}),\,{E}_{q,s}({{\rm{\Gamma }}}_{A|{B}_{j}})=0,$$

for *j* = *N*, …, 2^*n*^ − 1, and

29 $${{\rm{\Gamma }}}_{A{B}_{j}}={\rho }_{A{B}_{j}},$$

for each *j* = 0, …, *N* − 1. Thus, Inequality (27) together with Eqs (28) and (29) leads us to

30 $$\begin{array}{rcl}{({E}_{q,s}({\rho }_{A|{B}_{0}{B}_{1}\cdots {B}_{N-1}}))}^{\alpha } & = & {({E}_{q,s}({{\rm{\Gamma }}}_{A|{B}_{0}{B}_{1}\cdots {B}_{{2}^{n}-1}}))}^{\alpha }\\  & \ge  & \sum _{j=0}^{{2}^{n}-1}\,{\alpha }^{{\omega }_{H}(\overrightarrow{j})}{({E}_{q,s}({{\rm{\Gamma }}}_{A|{B}_{j}}))}^{\alpha }\\  & = & \sum _{j=0}^{N-1}\,{\alpha }^{{\omega }_{H}(\overrightarrow{j})}{({E}_{q,s}({\rho }_{A|{B}_{j}}))}^{\alpha },\end{array}$$

and this completes the proof.□

We also note that, for any *α* ≥ 1 and the Hamming weight ${\omega }_{H}(\overrightarrow{j})$ of the binary vector $\overrightarrow{j}=({j}_{0},\ldots ,{j}_{n-1})$, ${\alpha }^{{\omega }_{H}(\overrightarrow{j})}$ is greater than or equal to 1, therefore

31 $${({E}_{q,s}({\rho }_{A|{B}_{0}{B}_{1}\cdots {B}_{N-1}}))}^{\alpha }\ge \sum _{j=0}^{N-1}\,{\alpha }^{{\omega }_{H}(\overrightarrow{j})}{({E}_{q,s}({\rho }_{A|{B}_{j}}))}^{\alpha }\ge \sum _{j=0}^{N-1}\,{({E}_{q,s}({\rho }_{A|{B}_{j}}))}^{\alpha },$$

for any multi-qubit state ${\rho }_{A{B}_{0}{B}_{1}\cdots {B}_{N-1}}$ and *α* ≥ 1.

For the validity of Inequality (14) in multi-party higher-dimensional quantum systems more than qubits, let us consider the counterexample of CKW inequality in three-qutrit systems^20^

32 $${|\psi \rangle }_{ABC}=\frac{1}{\sqrt{6}}(|123\rangle -|132\rangle +|231\rangle -|213\rangle +|312\rangle -|321\rangle {)}_{ABC}.$$

Here we would like to remark that, for a selective choice of *q* and *s* as well as *α*, Inequality (14) still holds for the counterexample of CKW inequality in Eq. (32); we first note that the two-qutrit reduced density matrix *ρ*_*AB*_ of ${|\psi \rangle }_{ABC}$ in Eq. (32) has a spectral decomposition,

33 $${\rho }_{AB}=\frac{1}{3}{(|x\rangle }_{AB}{\langle x|+|y\rangle }_{AB}{\langle y|+|z\rangle }_{AB}\langle z|),$$

where

34 $$|x{\rangle }_{AB}=\frac{1}{\sqrt{2}}(|23\rangle -|32\rangle ),\,|y{\rangle }_{AB}=\frac{1}{\sqrt{2}}(|31\rangle -|13\rangle ),\,|z{\rangle }_{AB}=\frac{1}{\sqrt{2}}(|12\rangle -|21\rangle ).$$

By the Hughston-Jozsa-Wootters (HJW) theorem^30^, any pure state ensemble of *ρ*_*AB*_ can be realized as a superposition of ${|x\rangle }_{AB}$, ${|y\rangle }_{AB}$ and ${|z\rangle }_{AB}$. Moreover, it is also straightforward to check that for arbitrary pure states |*ϕ*〉_*AB*_ = *c*_1_|*x*〉_*AB*_ + *c*_2_|*y*〉_*AB*_ + *c*_3_|*z*〉_*AB*_ with |*c*_1_|^2^ + |*c*_2_|^2^ + |*c*_3_|^2^ = 1, its reduced density matrix *σ*_*A*_ = tr_*B*_|*ϕ*〉_*AB*_ 〈*ϕ*| has the same spectrum $\{\frac{1}{2},\frac{1}{2},0\}$. Thus we have

35 $${E}_{q,s}({\rho }_{A|B})={E}_{q,s}({|\varphi \rangle }_{A|B})={S}_{q,s}({\sigma }_{A}).$$

For the choice of *s* = 0 and *q* = 3, the unified-(*q*, *s*) entropy in Eq. (1) is reduced to ${S}_{3,0}(\rho )=-\,\frac{1}{2}\,\mathrm{log}\,{\rm{tr}}\,{\rho }^{3}$, thus we have ${E}_{3,0}({\rho }_{A|B})=-\,\frac{1}{2}\,\mathrm{log}\,{\rm{tr}}\,{\sigma }_{A}^{3}=1$, where the symmetry of ${|\psi \rangle }_{ABC}$ under the permutation of subsystems, regardless of the global phase, also guarantees *E*_3,0_(*ρ*_*A*|*C*_) = 1. (For calculation simplicity, we used the logarithmic function based on 2 throughout this paper, which does not affect on the validity of monogamy and polygamy inequalities). Now, we have

36 $${({E}_{3,0}({|\psi \rangle }_{A|BC}))}^{\alpha }={(\mathrm{log}3)}^{\alpha }\ge 1+\alpha ={({E}_{3,0}({\rho }_{A|B}))}^{\alpha }+\alpha {({E}_{3,0}({\rho }_{A|C}))}^{\alpha }$$

for any *α* ≥ 4, which shows that Inequality (14) still holds for the counterexample of CKW inequality in Eq. (32) for a selective choice of *q* and *s* as well as *α*.

We also note that an analogous argument can be made to show the validity of Inequality (14) for the other counterexample of CKW inequality in $3\otimes 2\otimes 2$ quantum systems^8^. Thus Theorem 2 provides us with a new class of tight monogamy inequalities of multi-qubit entanglement even without any concrete counterexample in higher-dimensional quantum systems more that qubits.

The following theorem shows that Inequality (14) of Theorem 2 can be even improved to be a tighter inequality with some condition on two-qubit entanglement;

#### **Theorem 3**.

*For α* ≥ 1, *q* ≥ 2, 0 ≤ *s* ≤ 1, *qs* ≤ 3 *and any multi-qubit state* ${\rho }_{A{B}_{0}\cdots {B}_{N-1}}$, *we have*

37 $${({E}_{q,s}({\rho }_{A|{B}_{0}\cdots {B}_{N-1}}))}^{\alpha }\ge \sum _{j=0}^{N-1}\,{\alpha }^{j}{({E}_{q,s}({\rho }_{A|{B}_{j}}))}^{\alpha },$$

*conditioned that*

38 $${E}_{q,s}({\rho }_{A|{B}_{i}})\ge \sum _{j=i+1}^{N-1}\,{E}_{q,s}({\rho }_{A|{B}_{j}}),$$

*for i* = 0, …, *N* − 2.

#### *Proof*.

Due to Inequality (16), it is enough to show

39 $${(\sum _{j=0}^{N-1}{E}_{q,s}({\rho }_{A|{B}_{j}}))}^{\alpha }\ge \sum _{j=0}^{N-1}\,{\alpha }^{j}{({E}_{q,s}({\rho }_{A|{B}_{j}}))}^{\alpha },$$

and we use mathematical induction on *N*. We further note that Inequality (20) in the proof of Theorem 2 assures that Inequality (39) is true for *N* = 2.

Now let us assume the validity of Inequality (39) for any positive integer less than *N*. For a multi-qubit state ${\rho }_{A{B}_{0}\cdots {B}_{N-1}}$, we have

40 $${(\sum _{j=0}^{N-1}{E}_{q,s}({\rho }_{A|{B}_{j}}))}^{\alpha }={({E}_{q,s}({\rho }_{A|{B}_{0}}))}^{\alpha }{(1+\frac{{\sum }_{j=1}^{N-1}{E}_{q,s}({\rho }_{A|{B}_{j}})}{{E}_{q,s}({\rho }_{A|{B}_{0}})})}^{\alpha },$$

where Inequality (12) and the condition in Inequality (38) lead Inequality (40) to

41 $${(1+\frac{{\sum }_{j=1}^{N-1}{E}_{q,s}({\rho }_{A|{B}_{j}})}{{E}_{q,s}({\rho }_{A|{B}_{0}})})}^{\alpha }\ge 1+\alpha {(\frac{{\sum }_{j=1}^{N-1}{E}_{q,s}({\rho }_{A|{B}_{j}})}{{E}_{q,s}({\rho }_{A|{B}_{0}})})}^{\alpha }.$$

Thus Eq. (40) and Inequality (41) imply

42 $$\begin{array}{rcl}{(\sum _{j=0}^{N-1}{E}_{q,s}({\rho }_{A|{B}_{j}}))}^{\alpha } & \ge  & {({E}_{q,s}({\rho }_{A|{B}_{0}}))}^{\alpha }+\alpha {(\sum _{j=1}^{N-1}{E}_{q,s}({\rho }_{A|{B}_{j}}))}^{\alpha }\\  & \ge  & \sum _{j=0}^{N-1}\,{\alpha }^{j}{({E}_{q,s}({\rho }_{A|{B}_{j}}))}^{\alpha },\end{array}$$

where the second inequality is due to the induction hypothesis, and this complete the theorem.□

For any nonnegative integer *j* and its corresponding binary vector $\overrightarrow{j}$, the Hamming weight ${\omega }_{H}(\overrightarrow{j})$ is bounded above by ${\mathrm{log}}_{2}\,j$. Thus we have

43 $${\omega }_{H}(\overrightarrow{j})\le {\mathrm{log}}_{2}\,j\le j,$$

which implies

44 $${({E}_{q,s}({\rho }_{A|{B}_{0}\cdots {B}_{N-1}}))}^{\alpha }\ge \sum _{j=0}^{N-1}\,{\alpha }^{j}{({E}_{q,s}({\rho }_{A|{B}_{j}}))}^{\alpha }\ge \sum _{j=0}^{N-1}\,{\alpha }^{{\omega }_{H}(\overrightarrow{j})}{({E}_{q,s}({\rho }_{A|{B}_{j}}))}^{\alpha },$$

for any *α* ≥ 1. In other words, Inequality (14) in Theorem 2 can be made to be even tighter as Inequality (37) of Theorem 3 for any multi-qubit state ${\rho }_{A{B}_{0}{B}_{1}\cdots {B}_{N-1}}$ satisfying the condition in Inequality (38).

### Tight polygamy constraints of multi-qubit entanglement in terms of unified entanglement of assistance

As a dual property to the Inequality (14) of Theorem 2, we provide a class of polygamy inequalities of multi-qubit entanglement in terms of powered UEoA.

#### **Theorem 4**.

*For* 0 ≤ *β* ≤ 1, −*q*^2^ + 4*q* − 3 ≤ *s* ≤ 1 *on* 1 ≤ *q* ≤ 2 *and any multi-qubit state* ${\rho }_{A{B}_{0}\cdots {B}_{N-1}}$, *we have*

45 $${({E}_{q,s}^{a}({\rho }_{A|{B}_{0}{B}_{1}\cdots {B}_{N-1}}))}^{\beta }\le \sum _{j=0}^{N-1}\,{\beta }^{{\omega }_{H}(\overrightarrow{j})}{({E}_{q,s}^{a}({\rho }_{A|{B}_{j}}))}^{\beta }.$$

#### *Proof*.

Without loss of generality, we assume the ordering of the qubit subsystems *B*_0_, …, *B*_*N*−1_ satisfying

46 $${E}_{q,s}^{a}({\rho }_{A|{B}_{j}})\ge {E}_{q,s}^{a}({\rho }_{A|{B}_{j+1}})\ge 0$$

for each *j* = 0, …, *N* − 2. Moreover, due to the monotonicity of the function *f*(*x*) = *x*^*β*^ for 0 ≤ *β* ≤ 1 and the UEoA-based multi-qubit polygamy inequality in (9), we have

47 $${({E}_{q,s}^{a}({\rho }_{A|{B}_{0}{B}_{1}\cdots {B}_{N-1}}))}^{\beta }\le {(\sum _{j=0}^{N-1}{E}_{q,s}^{a}({\rho }_{A|{B}_{j}}))}^{\beta },$$

thus it is enough to show that

48 $${(\sum _{j=0}^{N-1}{E}_{q,s}^{a}({\rho }_{A|{B}_{j}}))}^{\beta }\le \sum _{j=0}^{N-1}\,{\beta }^{{\omega }_{H}(\overrightarrow{j})}{({E}_{q,s}({\rho }_{A|{B}_{j}}))}^{\beta }.$$

The proof method is similar to that of Theorem 2; we first prove Inequality (48) for the case that *N* = 2^*n*^ by using mathematical induction on *n*, and generalize the result to any positive integer *N*. For *n* = 1 and a three-qubit state ${\rho }_{A{B}_{0}{B}_{1}}$ with two-qubit rduced density matrices ${\rho }_{A{B}_{0}}$ and ${\rho }_{A{B}_{1}}$, we have

49 $${({E}_{q,s}^{a}({\rho }_{A|{B}_{0}})+{E}_{q,s}^{a}({\rho }_{A|{B}_{1}}))}^{\beta }={({E}_{q,s}^{a}({\rho }_{A|{B}_{0}}))}^{\beta }{(1+\frac{{E}_{q,s}^{a}({\rho }_{A|{B}_{1}})}{{E}_{q,s}^{a}({\rho }_{A|{B}_{0}})})}^{\beta },$$

which, together with Inequalities (13) and (46) leads us to

50 $${({E}_{q,s}^{a}({\rho }_{A|{B}_{0}})+{E}_{q,s}^{a}({\rho }_{A|{B}_{1}}))}^{\beta }\le {({E}_{q,s}^{a}({\rho }_{A|{B}_{0}}))}^{\beta }+\beta {({E}_{q,s}^{a}({\rho }_{A|{B}_{1}}))}^{\beta }.$$

Inequality (50) recovers Inequality (48) for *n* = 1.

Now we assume the validity of Inequality (48) for *N* = 2^*n*−1^ with *n* ≥ 2, and consider the case that *N* = 2^*n*^. For an (*N* + 1)-qubit state ${\rho }_{A{B}_{0}{B}_{1}\cdots {B}_{N-1}}$ and its two-qubit reduced density matrices ${\rho }_{A{B}_{j}}$ with *j* = 0, …, *N* − 1, we have

51 $${(\sum _{j=0}^{N-1}{E}_{q,s}^{a}({\rho }_{A|{B}_{j}}))}^{\beta }={(\sum _{j=0}^{{2}^{n-1}-1}{E}_{q,s}^{a}({\rho }_{A|{B}_{j}}))}^{\beta }\,{(1+\frac{{\sum }_{j={2}^{n-1}}^{{2}^{n}-1}{E}_{q,s}^{a}({\rho }_{A|{B}_{j}})}{{\sum }_{j=0}^{{2}^{n-1}-1}{E}_{q,s}^{a}({\rho }_{A|{B}_{j}})})}^{\beta },$$

where the ordering of subsystems in Inequality (46) and Inequality (13) together with Eq. (51) lead us to

52 $${(\sum _{j=0}^{N-1}{E}_{q,s}^{a}({\rho }_{A|{B}_{j}}))}^{\beta }\le {(\sum _{j=0}^{{2}^{n-1}-1}{E}_{q,s}^{a}({\rho }_{A|{B}_{j}}))}^{\beta }+\beta {(\sum _{j={2}^{n-1}}^{{2}^{n}-1}{E}_{q,s}^{a}({\rho }_{A|{B}_{j}}))}^{\beta }.$$

Because each summation on the right-hand side of Inequality (52) is a summation of 2^*n*−1^ terms, the induction hypothesis assures that

53 $${(\sum _{j\mathrm{=0}}^{{2}^{n-1}-1}{E}_{q,s}^{a}({\rho }_{A|{B}_{j}}))}^{\beta }\le \sum _{j\mathrm{=0}}^{{2}^{n-1}-1}\,{\beta }^{{\omega }_{H}(\overrightarrow{j})}{({E}_{q,s}^{a}({\rho }_{A|{B}_{j}}))}^{\beta },$$

and

54 $${(\sum _{j={2}^{n-1}}^{{2}^{n}-1}{E}_{q,s}^{a}({\rho }_{A|{B}_{j}}))}^{\beta }\le \sum _{j={2}^{n-1}}^{{2}^{n}-1}\,{\beta }^{{\omega }_{H}(\overrightarrow{j})-1}{({E}_{q,s}^{a}({\rho }_{A|{B}_{j}}))}^{\beta }.$$

(Possibly, we may index and reindex subsystems to get Inequality (54), if necessary). Thus, Inequalities (52), (53) and (54) recover Inequality (48) when *N* = 2^*n*^.

For an arbitrary positive integer *N* and a (*N* + 1)-qubit state ${\rho }_{A{B}_{0}{B}_{1}\cdots {B}_{N-1}}$, we consider the (2^*n*^ + 1)-qubit state ${{\rm{\Gamma }}}_{A{B}_{0}{B}_{1}\cdots {B}_{{2}^{n}-1}}$ in Eq. (26). Because ${{\rm{\Gamma }}}_{A{B}_{0}{B}_{1}\cdots {B}_{{2}^{n}-1}}$ is a (2^*n*^ + 1)-qubit state, we have

55 $${({E}_{q,s}^{a}({{\rm{\Gamma }}}_{A|{B}_{0}{B}_{1}\cdots {B}_{{2}^{n}-1}}))}^{\beta }\le \sum _{j=0}^{{2}^{n}-1}\,{\beta }^{{\omega }_{H}(\overrightarrow{j})}{({E}_{q,s}^{a}({{\rm{\Gamma }}}_{A|{B}_{j}}))}^{\beta },$$

where ${{\rm{\Gamma }}}_{A{B}_{j}}$ is the two-qubit reduced density matric of ${{\rm{\Gamma }}}_{A{B}_{0}{B}_{1}\cdots {B}_{{2}^{n}-1}}$ for each *j* = 0, …, 2^*n*^ − 1.

Moreover, ${{\rm{\Gamma }}}_{A{B}_{0}{B}_{1}\cdots {B}_{{2}^{n}-1}}$ is a product state of ${\rho }_{A{B}_{0}{B}_{1}\cdots {B}_{N-1}}$ and ${\sigma }_{{B}_{N}\cdots {B}_{{2}^{n}-1}}$, which implies

56 $${E}_{q,s}^{a}({{\rm{\Gamma }}}_{A|{B}_{0}{B}_{1}\cdots {B}_{{2}^{n}-1}})={E}_{q,s}^{a}({\rho }_{A|{B}_{0}{B}_{1}\cdots {B}_{N-1}}),\,{E}_{q,s}^{a}({{\rm{\Gamma }}}_{A|{B}_{j}})=0,$$

for *j* = *N*, …, 2^*n*^ − 1, and

57 $${{\rm{\Gamma }}}_{A{B}_{j}}={\rho }_{A{B}_{j}},$$

for each *j* = 0, …, *N* − 1. Thus Inequality (55) together with Eqs (56) and (57) recovers Inequality (45), and this completes the proof.

To illustrate the tightness of Inequality (45) in Theorem 4, let us first recall the general polygamy inequality of entanglement in arbitrary-dimensional multi-party quantum systems^16^;□

58 $${E}^{a}({\rho }_{{A}_{1}|{A}_{2}\cdots {A}_{n}})\le \sum _{i=2}^{n}\,{E}^{a}({\rho }_{{A}_{1}|{A}_{i}}),$$

for any multi-party quantum state ${\rho }_{{A}_{1}{A}_{2}\cdots {A}_{n}}$, where *E*^*a*^(*ρ*_*A*|*B*_) is EoA of *ρ*_*AB*_ in Eq. (7).

Now let us consider the three-qubit W-state

59 $${|W\rangle }_{ABC}=\frac{1}{\sqrt{3}}{(|100\rangle +|010\rangle +|001\rangle )}_{ABC},$$

where $${E}^{a}({|W\rangle }_{A|BC})=S({\rho }_{A})=\,\mathrm{log}\,3-\frac{2}{3}$$ and the EoA of the two-qubit reduced density matrices are $${E}^{a}({\rho }_{A|B})=$$$${E}^{a}({\rho }_{A|C})=\frac{2}{3}$$^31^. Thus, the marginal EoA from Inequality (58) is

60 $${E}^{a}({\rho }_{A|B})+{E}^{a}({\rho }_{A|C})-{E}^{a}({\rho }_{A|BC})=2-\,\mathrm{log}\,3\approx \mathrm{0.415.}$$

For *q* tends to 1, the unified-(*q*, *s*) entanglement is reduced to EoA as in Eq. (7), therefore the marginal UEoA from Inequality (45) for three-qubit, W-state when *q* = 1 and $\beta =\frac{1}{3}$ or $\frac{1}{4}$ are

61 $$\begin{array}{rcl}{E}_{(1,s)}^{a}{({\rho }_{A|B})}^{1/3}+\frac{1}{3}{E}_{(1,s)}^{a}{({\rho }_{A|C})}^{1/3}-{E}_{(1,s)}^{a}{({\rho }_{A|BC})}^{1/3} & \approx  & 0.196,\\ {E}_{(1,s)}^{a}{({\rho }_{A|B})}^{1/4}+\frac{1}{4}{E}_{(1,s)}^{a}{({\rho }_{A|C})}^{1/4}-{E}_{(1,s)}^{a}{({\rho }_{A|BC})}^{1/4} & \approx  & \mathrm{0.154.}\end{array}$$

Thus Inequality (45) is generally tighter than Inequality (58), which also delivers better bounds to characterize the W-class type three-party entanglement by means of bipartite ones.

We further note that an analogous argument for the improvement of monogamy inequalities from Theorem 2 to Theorem 3 can also be applied to Inequality (45) of Theorem 4 for a tighter class of polygamy inequalities with some condition on two-qubit entanglement of assistance.

#### **Theorem 5**.

*For* 0 ≤ *β* ≤ 1, −*q*^2^ + 4*q* − 3 ≤ *s* ≤ 1 *on* 1 ≤ *q* ≤ 2 *and any multi-qubit state* ${\rho }_{A{B}_{0}\cdots {B}_{N-1}}$, *we have*

62 $${({E}_{q,s}^{a}({\rho }_{A|{B}_{0}\cdots {B}_{N-1}}))}^{\beta }\le \sum _{j=0}^{N-1}\,{\beta }^{j}{({E}_{q,s}^{a}({\rho }_{A|{B}_{j}}))}^{\beta },$$

*conditioned that*

63 $${E}_{q,s}^{a}({\rho }_{A|{B}_{i}})\ge \sum _{j=i+1}^{N-1}\,{E}_{q,s}^{a}({\rho }_{A|{B}_{j}}),$$

*for i* = 0, …, *N* − 2.

From Inequality (43), we have ${\omega }_{H}(\overrightarrow{j})\le j$ for any nonnegative integer *j* and its corresponding binary vector $\overrightarrow{j}$, therefore

64 $${({E}_{q,s}^{a}({\rho }_{A|{B}_{0}\cdots {B}_{N-1}}))}^{\beta }\le \sum _{j=0}^{N-1}\,{\beta }^{j}{({E}_{q,s}^{a}({\rho }_{A|{B}_{j}}))}^{\beta }\le \sum _{j=0}^{N-1}\,{\beta }^{{\omega }_{H}(\overrightarrow{j})}{({E}_{q,s}^{a}({\rho }_{A|{B}_{j}}))}^{\beta },$$

for 0 ≤ *β* ≤ 1. Thus, Inequality (62) of Theorem 5 is tighter than Inequality (45) of Theorem 4 for 0 ≤ *β* ≤ 1 and any multi-qubit state ${\rho }_{A{B}_{0}{B}_{1}\cdots {B}_{N-1}}$ satisfying the condition in Inequality (63).

## Discussion

Since its inception, understanding the nature of quantum entanglement is at the heart of quantum information theory. Although entanglement in bipartite quantum systems has been well studied with rich understanding, the situation becomes far more difficult for the case of multi-partite quantum entanglement, and only few are known for its characterization as well as its quantification. On the other hand, the saturation of monogamy and polygamy inequalities of multi-party entanglement provide us with an efficient way of characterizing multi-party quantum entanglements among different classes, because the genuine multi-party entanglement of this type can be completely characterized by means of the two-way (bipartite) entanglement within it. Thus it is an important task to have proper bipartite entanglement quantifications showing tight monogamy and polygamy inequalities for an efficient characterization of entanglements from different classes in multi-party and even in high-dimensional quantum systems.

Here we have provided a characterization of multi-qubit entanglement monogamy and polygamy constraints in terms of non-negative power of entanglement measures based on unified entropy. Using the Hamming weight of the binary vector related with the distribution of subsystems, we have established a class of monogamy inequalities of multi-qubit entanglement based on the *α*th-power of UE for *α* ≥ 1. We have also established a class of polygamy inequalities of multi-qubit entanglement in terms of the *β*th-power of UEoA for 0 ≤ *β* ≤ 1.

Our results deal with the full range of non-negative power of the most general class of bipartite entanglement measures based on unified-(*q*, *s*) entropy to establish monogamy and polygamy inequalities of multi-qubit entanglement, therefore our results encapsulate the results of various entropy-based monogamy and polygamy inequalities as special cases. For a selective choice of parameters *q*, *s* and *α*, our class of monogamy inequalities are also valid for the counterexamples of CKW monogamy inequality in higher-dimensional systems more than qubits.

We also remark that the class of monogamy and polygamy inequalities established here hold in a tighter way than other multi-qubit entanglement inequalities provided so far. Thus our results can provide an efficient way of characterizing entanglement shareability and distribution among the multi-party quantum systems without any known counterexample even in higher-dimensional systems more than qubits.
